# E-cigarette use and relations to tobacco and alcohol use among adolescents

**DOI:** 10.1186/s12916-015-0339-y

**Published:** 2015-04-30

**Authors:** Alfgeir L Kristjansson, Inga Dora Sigfusdottir

**Affiliations:** Department of Social and Behavioral Sciences, School of Public Health, West Virginia University, Morgantown, WV 26506 USA; Icelandic Center for Social Research and Analysis, Reykjavik University, 1 Menntavegur, Reykjavik, 101 Iceland; Department of Psychology, Reykjavik University, 1 Menntavegur, Reykjavik, 101 Iceland; Department of Health and Behavior Studies, Teachers College, Columbia University, New York City, NY 10027 USA

**Keywords:** Adolescents, Alcohol use, Delinquency, E-cigarettes, Electronic cigarettes, Smoking, Vapors

## Abstract

Electronic cigarette (EC) use is currently subject to a debate concerning safety, regulation need, and probable contribution to smoking cessation. An important gap in this debate is the lack of distinction between minors and adults. This is problematic because other principles of prevention apply to long-term users (such as most adult smokers) and experimental or probable users (more common in minors). This commentary focuses on two less discussed aspects of the EC debate: 1) whether EC use is likely to be additive to conventional cigarette and other tobacco use among minors, and 2) if EC use is likely to contribute to an overall increase in alcohol consumption and other drug use among minors. We find the results by Hughes et al. and others indeed suggestive of both. We conclude that EC use is likely to be additive to other tobacco use and increase the risk for alcohol use, therefore serving as another potential route to hazard for even mildly risk-prone minors. Policies to restrict the access and use of EC among minors are encouraged.

Please see related article: http://www.biomedcentral.com/1471-2458/15/244

## Background

Tobacco, alcohol, and other substance use should be discouraged in minors. This view has close to universal recognition. Among recent trends in the area of smoking are the so called electronic cigarettes (ECs), which have largely been marketed as a safer alternative to conventional cigarette (CC) smoking [1,2], often with emphasis on young consumers [3]. EC use is currently subject to a heated debate concerning safety, regulation need, and probable contribution to smoking cessation [4-7]. Although much evidence has yet to be revealed with regards to the harm of e-cigarette use, it is not debated, from a purely toxicological point of view, that they are safer than combustible tobacco [5,8]. However, an important gap in this debate is the lack of distinction between minors and adults. This is problematic because other principles of prevention apply to long-term users (such as most adult smokers) and experimental or probable users (more common in minors) [9]. Taking account of this problem, this commentary focuses on two less discussed aspects of the EC debate: 1) whether EC use is likely to be additive to CC and other tobacco use among minors, and 2) if EC use is likely to contribute to an overall increase in alcohol consumption and other drug use among minors.

### Is EC use likely to be additive to CC and other tobacco use among minors?

Advocates for the public health impact of ECs mainly build their argument on the notion of smoking cessation, namely that ECs are less harmful to the user than combustible tobacco and may therefore make a suitable alternative for those who have repeatedly but unsuccessfully attempted to quit smoking [4]. This assertion rests on the pillars of tertiary prevention and the concept of harm reduction [10]. That is, helping those who already are heavy smokers curb their use and/or minimize harm. However, this viewpoint does rarely apply in the case of minors where primary prevention approaches are more appropriate. A critical question in this respect is whether EC use is additive to CC and other tobacco use among minors, therefore potentially serving to increase the overall prevalence of tobacco use in the population. Some of the findings by Hughes et al. [11] are undeniably suggestive of this. For example, 4.9% of all participants that had never smoked CCs had accessed ECs, which sums to be almost 16% of those that had accessed ECs. Additionally, 22.6% of those who had “tried CC but didn’t like it” had accessed ECs. Evidence from elsewhere are reminiscent of similar trends. Recent US-based studies show a substantial increase in lifetime EC use among minors in grades 6 to 12 during the last 2 to 3 years [12,13]. A study among 15- to 19-year-old adolescents in Poland showed an increase in lifetime EC use of 5.5% to almost 30% between 2011 and 2014 [14]. Further, new unpublished data from Iceland collected among 10^th^ grade students in February 2015, shows that 17.1% of students had tried ECs in their lifetime and 9.3% of those who had never used CCs had tried ECs. Another US-based study specifically examined alternate tobacco use in EC, CC, and non-users, and found that EC-only users were significantly more likely to use blunts and hookah than CC-only users [15]. Together, these findings strongly indicate that the presence of ECs serves to increase the overall rate of minors engaging in tobacco use (of any kind) and, therefore, that ECs are additive to the overall tobacco prevalence in the population among minors.

### Is EC use likely to increase the risk of alcohol consumption and other drug use among minors?

For several decades one of the most influential theories of primary prevention has been the “gateway hypothesis”. The central claim of the gateway hypothesis is that substance use and abuse can be lined up in a consequential trajectory of potential harm [16,17]. It further states that early initiation of CC use is likely to increase the risk of alcohol use, particularly among minors, and may then lead to increased risks of cannabis experimentation and use, which can lead to stronger substances [18,19]. This hypothetical sequence of substance use risk is especially important in minors because we know that for every year delayed in initiation of use the risk of consistent use and associated social developmental problems is substantially decreased [20-23]. This is the central assertion of primary substance use prevention. With regards to ECs, the critical question becomes what role, if any, EC use is likely to play in this hypothetical sequence, therefore potentially increasing the risk of overall alcohol use and other substance use among minors in the population? Some of the findings by Hughes et al. [11] do indeed point in this direction. For example, over 9% of non-drinkers had accessed ECs, and despite statistically controlling for smoking behaviors, regular alcohol users and binge drinkers were significantly more likely to have accessed ECs. Findings from other studies show similar results. A US-based study among young adolescents showed that EC users were significantly more likely than non-users to use both CC and alcohol [24]. Another study, conducted in 2013 in Hawaii, showed that EC-only users (as opposed to CC only or dual users) where significantly worse off on traditional risk and protective factors of primary prevention (e.g., parental support, academic involvement, peer smoking, etc.) than non-users but better off compared to dual or CC-only users [13]. The authors concluded that, with regards to risk and protective factors for substance use development, EC-only users fall in between non-users and CC/dual users as an intermediate group [13]. The new and unpublished data from 10^th^ grade students in Iceland mentioned earlier shows the exact same pattern: when non-users, CC-only, EC-only, and dual users are cross-tabulated on lifetime alcohol use and drunkenness, the prevalence of use by EC-only users falls in between non-users (lowest prevalence), CC-only users (second highest category), and dual users (highest prevalence). Together, the findings by Hughes et al. [11] and the latest research in the area therefore suggests that EC use among minors may serve to intensify the risk of other substance use, such as alcohol use, which in turn is likely to escalate into other substance use.

## Conclusions

Decades of prevention efforts have informed our children that smoking and other tobacco use is harmful for one’s health – this is essentially common sense in today’s Western world. However, it is not the case with ECs, which is likely to confuse minors about what is tolerable for their physical health and social development. Recent studies have shown that non-cigarette smoking and cigarette smoking minors believe ECs to be less harmful than CCs [3,25]. This will, without a doubt, serve to increase the odds of non-cigarette smoking minors engaging in EC use although they might never have considered using CCs. The current evidence of EC use by never, previous, and current smokers among minors suggests that EC use is additive to other tobacco use and increases the risk for alcohol use, therefore serving as yet another route to potential hazard for even mildly risk-prone minors. Preventive efforts at multiple levels to restrict the access and use of ECs among minors are therefore encouraged.

## Abbreviations

CCs: Conventional cigarettes
ECs: Electronic cigarettes
